# ATG9B regulates bacterial internalization via actin rearrangement

**DOI:** 10.1016/j.isci.2024.109623

**Published:** 2024-04-10

**Authors:** Junpei Iibushi, Takashi Nozawa, Hirotaka Toh, Ichiro Nakagawa

**Affiliations:** 1 Department of Microbiology, Graduate School of Medicine, Kyoto University, Yoshida-Konoe-cho, Sakyo-ku 606-8501, Kyoto, Japan

**Keywords:** Microbiology, Cell biology

## Abstract

Invasive bacterial pathogens are internalized by host cells through endocytosis, which is regulated by a cascade of actin rearrangement signals triggered by host cell receptors or bacterial proteins delivered into host cells. However, the molecular mechanisms that mediate actin rearrangement to promote bacterial invasion are not fully understood. Here, we show that the autophagy-related (ATG) protein ATG9B regulates the internalization of various bacteria by controlling actin rearrangement. *ATG* knockout screening and knockdown experiments in HeLa cells identified ATG9B as a critical factor for bacterial internalization. In particular, cells with *ATG9B* knockdown exhibited an accumulation of actin filaments and phosphorylated LIM kinase and cofilin, suggesting that ATG9B is involved in actin depolymerization. Furthermore, the kinase activity of Unc-51-like autophagy-activating kinase 1 was found to regulate ATG9B localization and actin remodeling. These findings revealed a newly discovered function of ATG proteins in bacterial infection rather than autophagy-mediated immunity.

## Highlights

• ATG9B regulates bacterial internalization via actin rearrangement in Epithelial cells
• ATG9B modulates actin rearrangement through the phosphorylation cascade of LIMK/cofilin
• ULK1 is involved in bacterial internalization through the regulation of ATG9B localization

## Graphical abstract

**Figure:**
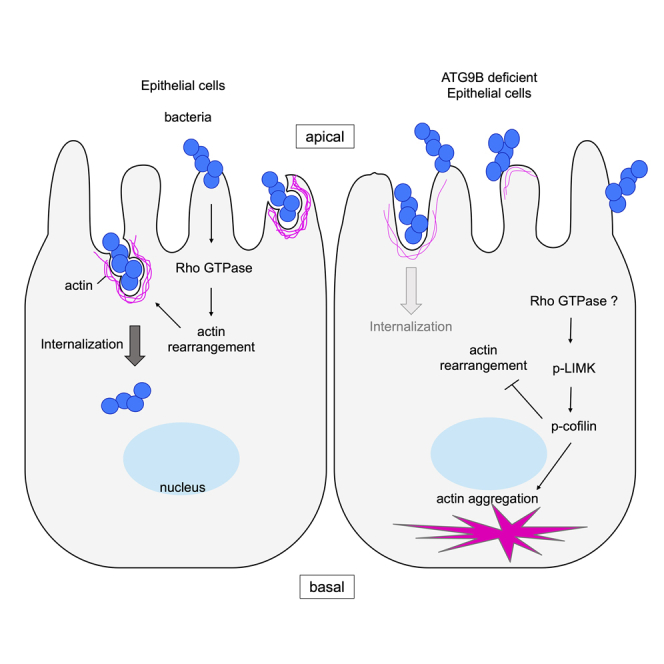

## Introduction

Many invasive bacterial pathogens manipulate the host actin cytoskeleton to enter non-phagocytic cells or to avoid phagocytosis by macrophages. Bacterial pathogens have evolved strategies to adhere to host cells (particularly non-phagocytic host cells) that involve inducing their phagocytosis.^1^ For instance, *Listeria monocytogenes* adheres by interacting with the E-cadherin or c-Met, surface proteins of host cells.^2^^,^^3^ Alternatively, *Yersinia pseudotuberculosis*, *Staphylococcus aureus*, and *Streptococcus pyogenes* (Group A *streptococcus*; GAS) express an extracellular matrix-binding protein on their outer membrane that facilitates internalization via integrin receptors.^4^^,^^5^^,^^6^ The interaction between this bacterial protein and the host cell receptor triggers a cascade of actin rearrangement signals that induce endocytosis. Other pathogens, such as *Shigella flexineri* and *Salmonella enterica* serovar Typhimurium, inject virulence factor proteins into host cells through a needle-like structure encoded by type III secretion systems, modulating intracellular trafficking and provoking the reorganization of the actin cytoskeleton for engulfment.^7^^,^^8^^,^^9^ Thus, bacterial pathogens commonly establish the internalization of host cells by targeting actin rearrangement.

The family of small Rho GTPases, which comprise >20 members in mammals, are critical regulators of actin cytoskeletal rearrangements; of these, RhoA, Rac1, and Cdc42 are the best studied.^10^^,^^11^ They cycle between GDP-bound (inactive) and GTP-bound (active) states, transducing extracellular signals to downstream effectors in the GTP-bound state. RhoA forms stress fibers and focal adhesion complexes, Rac1 triggers lamellipodia and membrane ruffling, and Cdc42 induces filopodia formation.^12^ During bacterial infection, these Rho GTPases are essential for intracellular internalization. For example, RhoA is critical for the uptake of *Mycobacterium avium* and *Pseudomonas aeruginosa*, whereas Rac1 and Cdc42 have crucial roles in the internalization of *S. flexineri* and *S. enterica* Typhimurium.^13^^,^^14^^,^^15^^,^^16^

Autophagy-related (ATG) genes are essential for inducing macroautophagy (hereafter referred to as autophagy), a widely conserved intracellular degradation mechanism contributing to cellular homeostasis. Autophagy induced by various stressors, such as nutrient starvation, pathogen invasion, and organelle damage, initially involves the formation of cup-shaped membrane structures called phagophores in the cytoplasm. Subsequently, the phagophores elongate to form a double membrane structure called an autophagosome that surrounds substrates targeted for subsequent delivery to lysosomes.^17^^,^^18^ This process dramatically changes the dynamics of intracellular membranes, and the formation of autophagosomes occurs through the cooperative action of an ATG protein interaction network.

Recent discoveries have revealed that ATG proteins are also involved, individually or as a functional group, in pathways distinct from autophagy, such as phagocytosis, apoptosis, and protein secretion. For example, LC3-associated phagocytosis is induced by phagocytic uptake of microbes. This pathway is not dependent on Unc-51-like autophagy-activating kinase (ULK)1, which is required for canonical autophagy and is not affected by starvation or rapamycin^19^. ATG16L1 promotes membrane repair and restricts bacterial spread,^20^ whereas ATG13 stimulates interferon expression and activates the JAK-STAT cascade during viral infection.^21^ However, although ATG proteins are known to mediate the regulation of intracellular membrane dynamics and form autophagosomes, no studies have reported on their involvement in the regulation of actin rearrangement.

In this study, we aimed to uncover non-autophagy induction-related physiological functions of ATG proteins by the examination of bacterial internalization during GAS infection in HeLa ATG gene knockout (KO) cells. We found that ATG9AB KO cells showed a decreased GAS internalization rate, and identified ATG9B as a regulator of actin rearrangement via LIM kinase (LIMK)/cofilin phosphorylation. Furthermore, the actin rearrangement-mediated regulation of several types of bacterial internalization was found to involve ULK1 kinase activity.

## Results

### *ATG9B* is involved in the internalization of group A *streptococcus*

**Figure 1:**
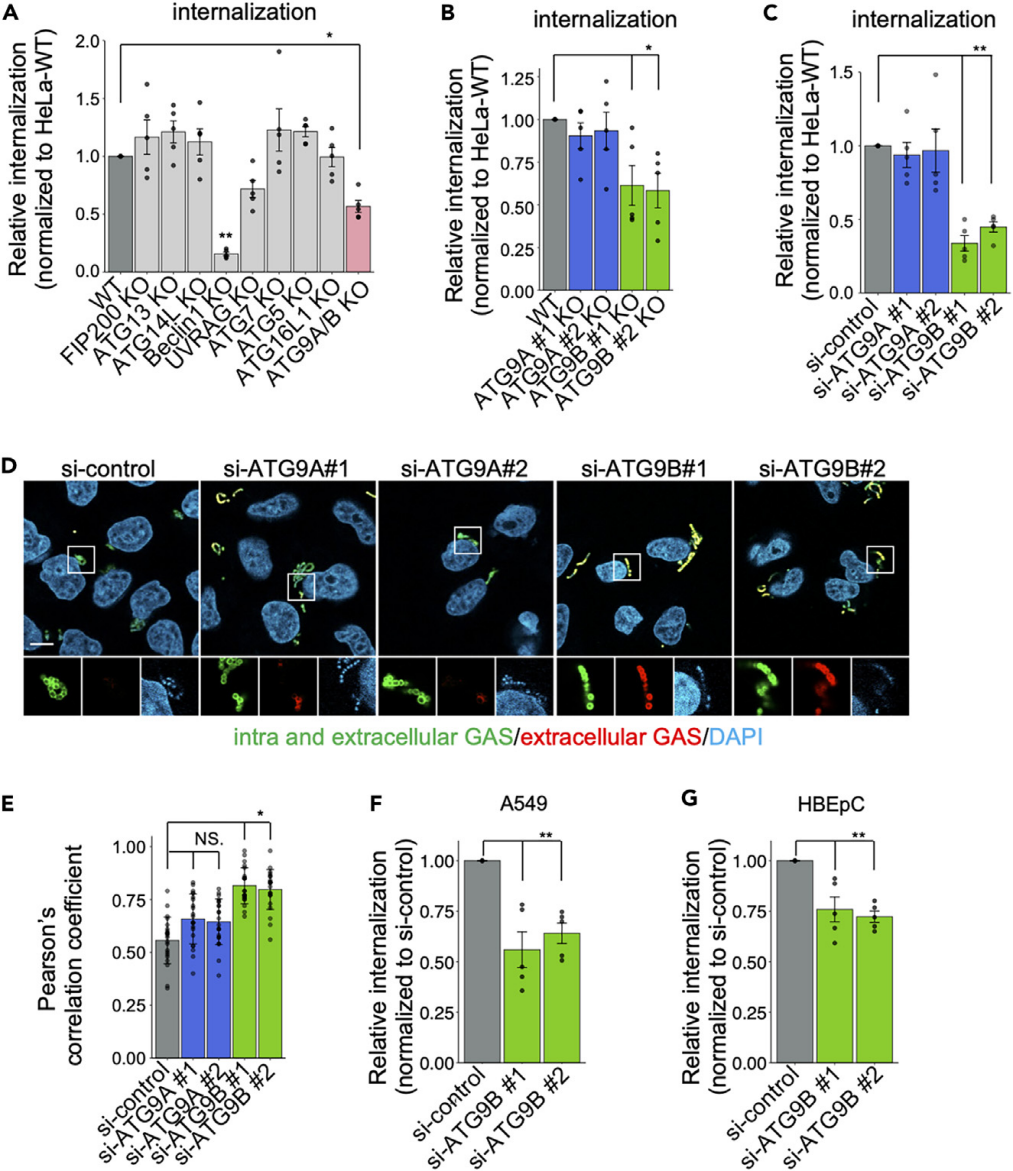
ATG9 is involved in the internalization of bacteria during GAS infection (A and B) GAS internalization in HeLa cell strains infected with Kos of various genes, including *ATG*, *ATG9A*, and *ATG9B*, at an MOI of 10. The relative percent internalization was normalized with data from WT HeLa cells (colony-forming units [CFU] recovered at 2 h post-infection [hpi]/CFUs at 1 hpi). (C) GAS internalization in HeLa cells treated with ATG9A- or ATG9B-targeted siRNA and infected at an MOI of 10. The relative percent internalization was normalized with data from non-targeting control siRNA-treated cells (CFUs recovered at 2 h hpi/CFUs at 1 hpi). (D) Immunostained extracellular GAS and total GAS (intracellular + extracellular GAS) in ATG9A or ATG9B siRNA-treated cells at 1 hpi. Extracellular GAS was labeled with Alexa Fluor 594, and total GAS was labeled with Alexa Fluor 488 after Triton X-100 permeabilization. Bacterial and cellular DNAs were stained with DAPI. (E) Quantification of the co-localization of extracellular GAS (Alexa Fluor 488) and total GAS (Alexa Fluor 594). Pearson’s correlation coefficients were quantified from 10 micrograph images per independent experiment using ImageJ/Fiji software. (F and G) GAS internalization in A549 cells (F) or HBEpCs (G) treated with ATG9B-targeted siRNA and infected at an MOI of 10. The relative percent internalization was normalized with data from non-targeting control siRNA-treated cells (CFUs recovered at 2 hpi/CFUs at 1 hpi).Scale bar, 10 μm. Data represent the mean ± SEM of > five independent experiments. Statistical analysis was performed using one-way analysis of variance (ANOVA), followed by Dunnett’s test or Tukey’s multiple comparison test; ∗*p* < 0.05, ∗∗*p* < 0.01.

To investigate whether ATG proteins are involved in the endocytosis of bacteria in non-phagocytic host cells, we generated a new KO of *ATG9A/B* in HeLa cells using CRISPR/Cas9 genome editing (Figures S1A and S1B). Each ATG KO strain was infected with GAS and then subjected to a gentamicin protection assay, which has been a commonly used method to assess bacterial internalization.^22^^,^^23^^,^^24^^,^^25^ Internalization was then defined as the number of colony-forming units (CFUs) at 2 h post-infection (hpi)/CFU at 1 hpi (Figures 1A, S2A, and S2B). Consistent with previous reports that KOs of *Beclin-1* or UV irradiation resistance-associated gene (*UVRAG*) inhibited bacterial invasion,^26^^,^^27^^,^^28^ we found that KO of *ATG9A/B* also decreased bacterial internalization (Figure 1A). In mammalian cells, *ATG9* has two homologs: *ATG9A* and *ATG9B*.^29^ To investigate the involvement of each of these genes in GAS internalization, we repeated the gentamicin protection assay in *ATG9A* and *ATG9B* KO cells. The GAS internalization rate decreased in *ATG9B* KO cells, but not in *ATG9A* KO cells (Figures 1B, S2C, and S2D), suggesting that only *ATG9B* is required for GAS invasion. We also performed knockdown (KD) of *ATG9A* and *ATG9B* expression using small interfering RNA (siRNA) and non-targeting RNA as a control (si-control). We found that the KD of *ATG9B*, but not that of *ATG9A*, substantially reduced GAS internalization (Figures 1C, S1D, S1E, S2E, and S2F). This phenotype may be less likely to occur in KO cells than in KD cells because a protein with a similar function may take the place of the protein that has been knocked out. Therefore, we decided to use KD cells. To confirm the reduced internalization of GAS in *ATG9B* KD cells, we further investigated GAS invasion using a differential immunostaining assay. Extracellular GAS was stained with Alexa Fluor 594 (red), while extracellular and intracellular GAS were stained with Alexa Fluor 488 (green). Therefore, extracellular GAS is visualized as red or yellow because of the co-localization of red and green, whereas intracellular GAS is only visualized as green. Quantification of the co-localization rate of red and green signals was higher when extracellular GAS was abundant; i.e., *ATG9B* KD cells had higher co-localization rates than si-control-treated cells (Figures 1D and 1E). This finding suggested that the internalization of GAS was indeed inhibited by *ATG9B* KD. To further characterize the *ATG9B* KD-mediated reduction of GAS internalization, we evaluated it in two other cell lines: A549, an alveolar epithelial cell line, and HBEpC, a bronchial epithelial cell line. Our results confirmed that KD of *ATG9B* also reduced GAS internalization in these cell lines (Figures 1F, 1G, S1E, S1F, and S2G–S2J), demonstrating that *ATG9B* is required to internalize GAS into epithelial cells.

### ATG9A, but not ATG9B, is essential for autophagy induction during group A *streptococcus* infection

**Figure 2:**
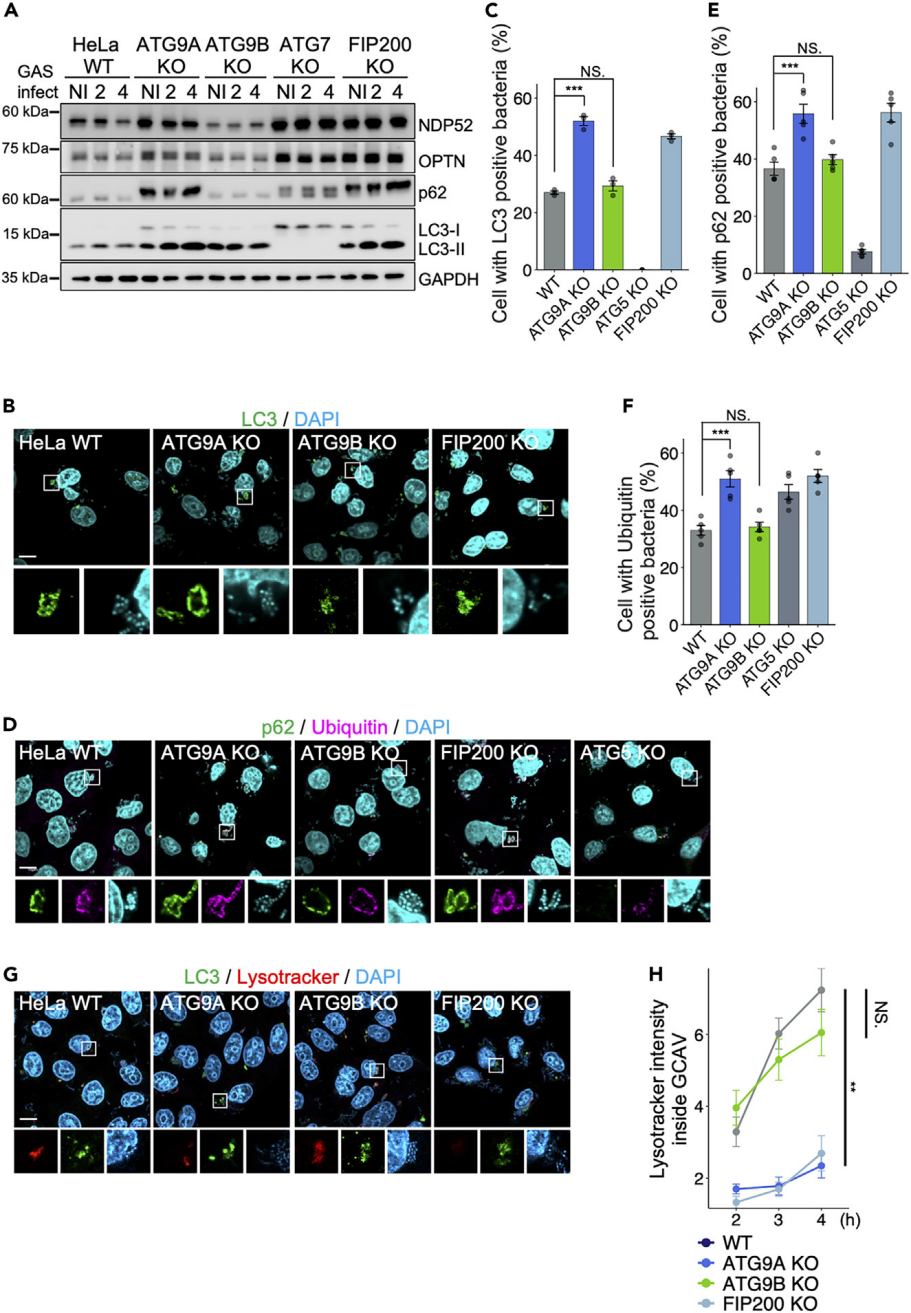
ATG9A is essential for autophagy induction during GAS infection (A) Immunoblotting analysis of various GAS-infected HeLa KO strains using the indicated antibodies. NI: non-infection. (B–F) Immunostaining of the indicated HeLa KO strains infected with GAS for 4 h, then fixed and incubated with LC3 (B), p62 (green), and ubiquitin (magenta) (D). Host cellular and bacterial DNA was stained with DAPI (cyan). Representative microscopic images (b, d) and quantification (C, E, F) of host cells with GAS positivity for the indicated antibodies. (G and H) HeLa KO strains infected with GAS (2, 3, or 4 hpi) and stained with LysoTracker red 30 min before fixation and with LC3 (green) after fixation. Representative microscopic images at 4 hpi (G) and quantification (H) of the LysoTracker intensity inside GAS-containing autophagosome-like vacuoles (GcAVs). The y axis shows the LysoTracker intensity in GcAvs, with higher values indicating lower pH.Data in C, E, F (*n* = 100 cells per condition), and H (*n* = 35 LC3-positive GAS) represent the mean ± SEM of > five independent experiments. Scale bar, 10 μm. One-way ANOVA was performed and *p* values were calculated using Tukey’s multiple comparison test; ∗*p <* 0.05, ∗∗*p <* 0.01, ∗∗∗*p <* 0.001.

ATG9 is one of the core proteins that induce canonical autophagy.^30^ To investigate the involvement of ATG9A and ATG9B in autophagy induction during GAS infection, we detected the autophagy marker LC3 and autophagy receptor proteins NDP52, OPTN, and p62 using western blotting in KO cells of *ATG9A* and *ATG9B* and, as negative controls, *ATG7*, a critical component of the LC3 conjugation system, and *FIP200*, which is dispensable for LC3 recruitment but essential for bacterial degradation.^31^ When autophagy flux is arrested, LC3-II and receptor proteins accumulate because they are not degraded by autophagy. Specifically regarding p62, which is phosphorylated to increase its affinity for the cargo,^32^^,^^33^^,^^34^ reduced autophagy flux leads to the accumulation of phosphorylated p62 and thus two identifiable bands for this protein. Indeed, we found that LC3-II and autophagy receptor proteins accumulated, and phosphorylated p62 bands were identified in KO cells of *ATG9A*, *ATG7*, and *FIP200*, but not in *ATG9B* KO cells, with or without GAS infection (Figures 2A and S3). Additionally, we observed the recruitment of LC3, p62, and ubiquitin to the bacteria at 3 h post-infection. LC3, p62, and ubiquitin recruitment were increased in *ATG9A* KO and *FIP200* KO cells compared to in *ATG9B* KO and wild-type (WT) HeLa cells (Figures 2B–2F). These results implied that KO of *ATG9A* or *FIP200* impaired autophagy flux. Next, we examined the acidification within GAS-containing autophagosome-like vacuoles (GcAVs) using LysoTracker. HeLa WT and *ATG9B* KO cells exhibited time-dependent, increased LysoTracker intensity inside GcAVs following GAS infection, whereas *ATG9A* KO and *FIP200* KO cells did not (Figures 2G and 2H). These results suggested that ATG9A is essential for the autophagy flux during GAS infection in HeLa cells, whereas ATG9B is dispensable.

### ATG9B regulates bacterial internalization via actin rearrangement

**Figure 3:**
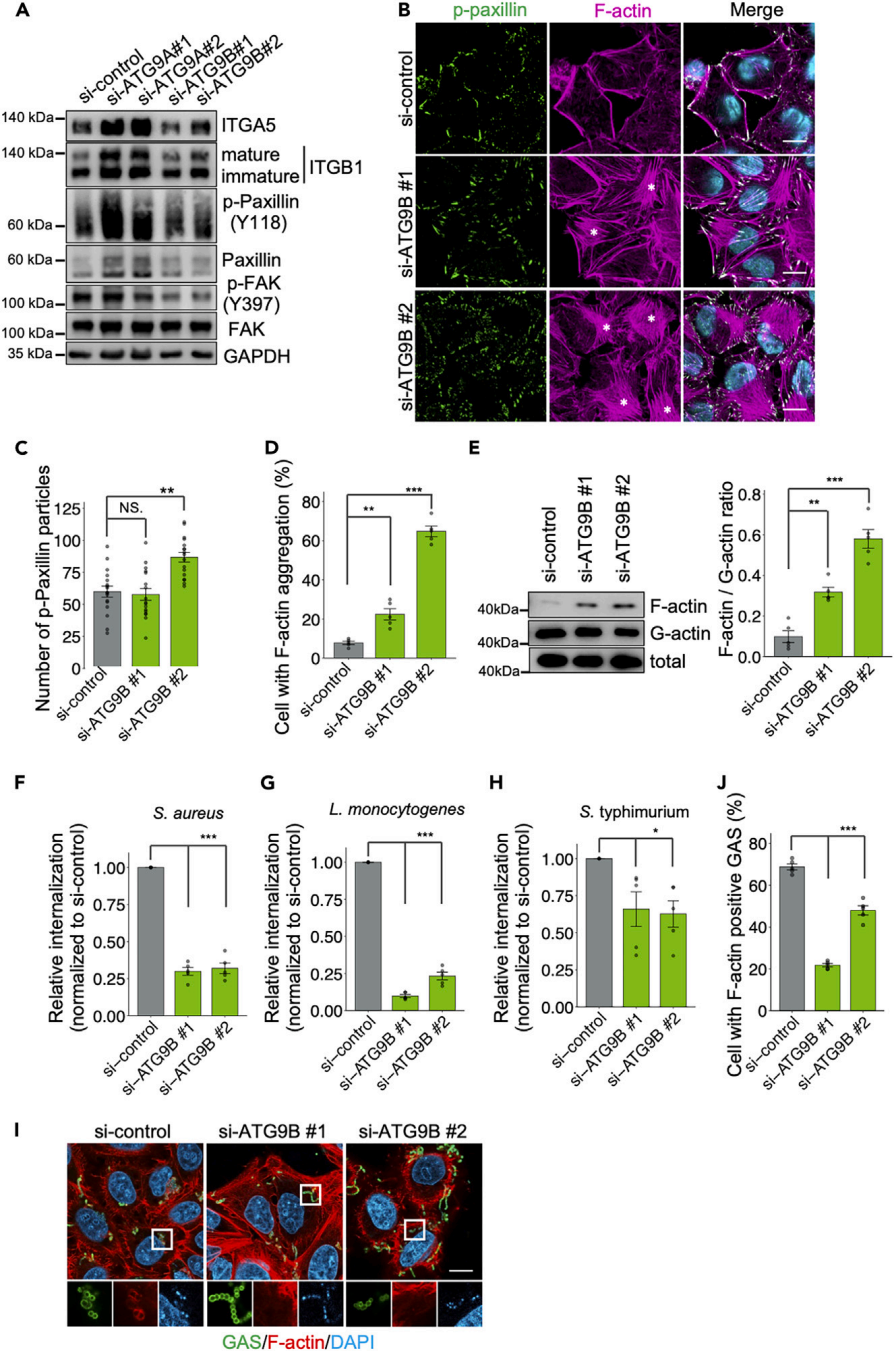
ATG9B KD cells form F-actin aggregates and control invasion by several bacterial species (A) Immunoblotting analysis of ATG9A or ATG9B siRNA-treated HeLa cells using the indicated antibodies. (B–D) Immunostaining of fixed ATG9B siRNA-treated HeLa cells for *p*-Paxillin (green) and F-actin (magenta). Cellular and bacterial DNA was stained with DAPI (cyan). Asterisks indicate actin aggregation. Representative microscopic images (B) and quantification of cells for the number of *p*-Paxillin particles (C) and percentage with F-actin aggregation (D). (E) Immunoblot analysis of F-actin and G-actin in ATG9B siRNA-treated HeLa cells with quantification using ImageJ/Fiji. (F–H) Internalization of the indicated bacteria by ATG9B siRNA-treated HeLa cells infected at an MOI of 10. The relative percent internalization was normalized with data from non-targeting control siRNA-treated cells (colony-forming units [CFU] recovered at 2 h post-infection [hpi]/CFUs at 1 hpi). (I and J) ATG9B siRNA-treated HeLa cells infected with GAS for 2 h and stained with anti-GAS antibody (green) and F-actin (red). Cellular and bacterial DNA was stained with DAPI (cyan). Representative microscopic images (I) and quantification of cells containing F-actin positive GAS (J). Data in C (*n* = 200 cells per condition), D (*n* = 100 cells per condition), and E–H represent the mean ± SEM from five independent experiments. Scale bar, 10 μm. One-way ANOVA was performed and *p* values were calculated using Tukey’s multiple comparison test; ∗*p <* 0.05, ∗∗*p <* 0.01, ∗∗∗*p <* 0.001.

GAS invades epithelial cells via the integrin α5β1 endocytotic pathway,^5^ and ATG9B promotes integrin β1 (ITGB1) polarization to the membrane to reassemble focal adhesions.^5^^,^^35^ ITGB1 is glycosylated in the endoplasmic reticulum to generate a partially glycosylated form, immature ITGB1, and then transported to the Golgi complex, where it is further glycosylated to mature ITGB1. It has been shown that mature ITGB1 is transported to the cell surface with integrin α subunits.^36^^,^^37^^,^^38^ To investigate how ATG9B regulates GAS internalization, we examined the expression level and localization of endogenous integrin α5β1. We did not observe any change in the expression level or localization of the integrin α5 and β1 subunits under KD of *ATG9A* or *ATG9B* (Figures 3A and S4A–S4D). Subsequently, we checked the expression levels of phosphorylated paxillin (*p*-paxillin) and phosphorylated FAK (*p*-FAK). Since integrin activates FAK by promoting its recruitment and autophosphorylation at Y397,^39^^,^^40^ and paxillin is phosphorylated at Y118 by FAK,^41^ we hypothesized that an impact of *ATG9* KD on integrin would also affect the phosphorylation of FAK and paxillin. However, we found no changes in the phosphorylation of FAK and paxillin in *ATG9A* and *ATG9B* KD cells (Figure 3A, S4E, and S4F). Next, we observed the localization of paxillin and actin filaments (F-actin) by confocal microscopy. The morphology of HeLa cells under KD with ATG9B siRNA #1 was spiky, and the localizations of paxillin and F-actin were pericellular (Figure 3B). In contrast, cells under ATG9B siRNA #2 KD had more paxillin particles and aggregated F-actin than control siRNA cells (Figures 3B–3D). To examine actin polymerization, we detected F-actin and actin monomers (G-actin) in these cells by western blotting. KD of *ATG9B* with either siRNA significantly increased the F-actin/G-actin ratio (Figure 3E). Collectively, these results demonstrate that *ATG9B* is involved in actin rearrangement.

Actin rearrangement is a common and essential process in the invasion of various pathogenic bacteria.^1^ Therefore, we evaluated whether ATG9B is also involved in bacterial internalization other than GAS. Indeed, *ATG9B* KD reduced the internalization rates of *S. aureus*, *L. monocytogenes*, and *S. enterica* Typhimurium (Figures 3F–3H), but its effect was least apparent on *S. enterica* Typhimurium. Hypothesizing that this result was due to the effectors of *S. enterica* Typhimurium that depolymerize actin,^42^^,^^43^ we examined the role of actin in bacterial recruitment during GAS infection. *ATG9B* KD cells showed reduced actin recruitment compared to si-control-treated cells (Figures 3I and 3J). Actin aggregation under KD of *ATG9B* may prevent the recruitment of actin, which is important for bacterial endocytosis. These findings suggest that *ATG9B* may regulate bacterial internalization through actin rearrangement.

### ATG9B modulates actin rearrangement through the LIM kinase/cofilin axis

**Figure 4:**
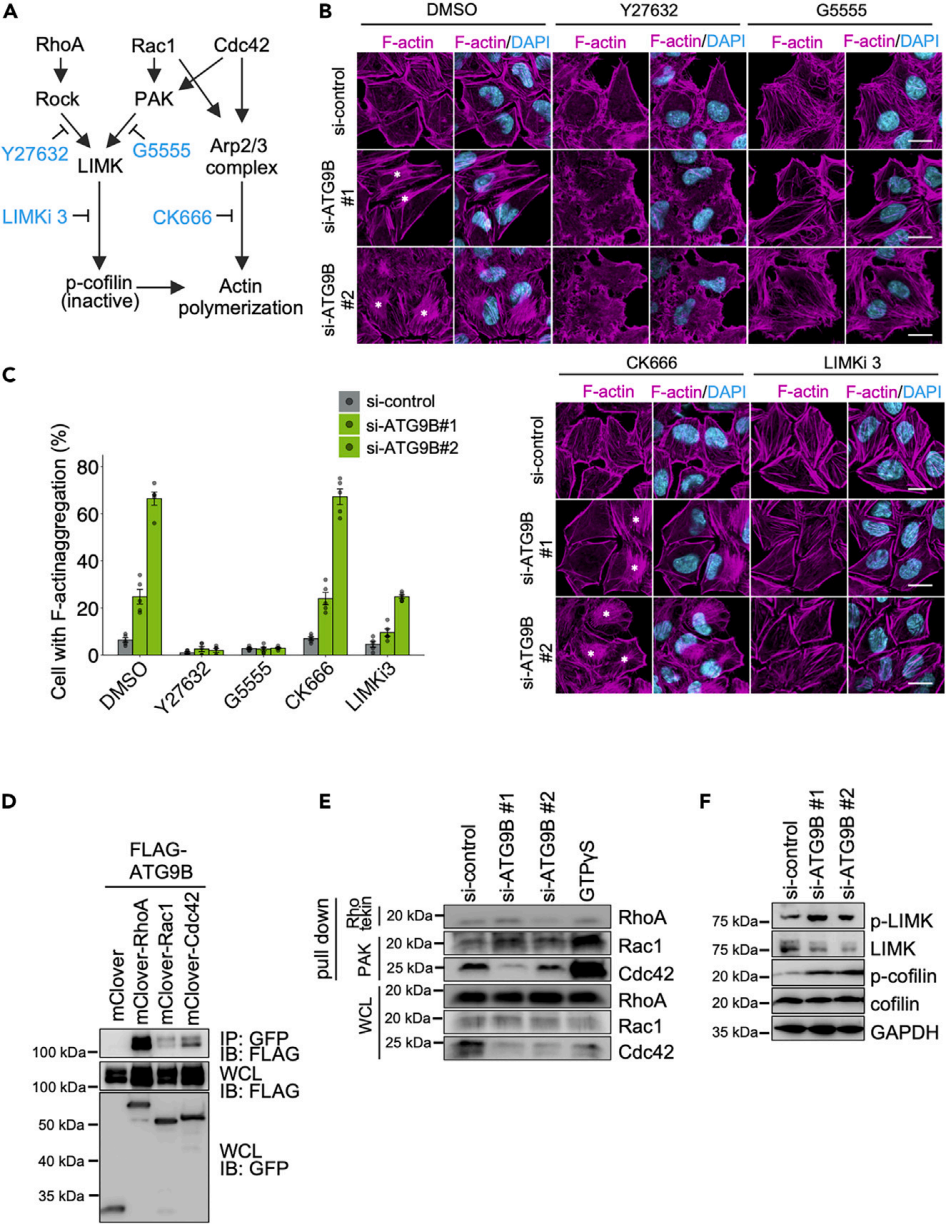
ATG9B modulates actin rearrangement in HeLa cells (A) Schematic representation of the RhoGTPase pathway and the pathways blocked by the respective inhibitors. (B and C) Treatment of ATG9B siRNA-treated HeLa cells with ROCK inhibitor (Y27632), PAK inhibitor (G5555), LIMK inhibitor LIMKi 3, or Arp2/3 complex inhibitor (CK666) for 2 h. Cells were fixed and immunostained for F-actin (magenta), and cellular DNA was stained with DAPI (cyan). Asterisks indicate actin aggregation. Representative microscopic images (B) and quantification of cell percentage with F-actin aggregation (C). (D) Co-IP between FLAG-tagged ATG9B and mClover-tagged Rho GTPase proteins. (E) Immunoblotting of Rho-binding domain or p21-binding domain agarose-isolated active RhoA, Rac1, or Cdc42 from whole cell lysates (WCL) obtained from ATG9B siRNA-treated HeLa cells. (F) Immunoblotting analysis of ATG9B siRNA-treated HeLa cells with the indicated antibodies.Data in c (*n* = 100 cells per condition) represent the mean ± SEM from five independent experiments: scale bar, 10 μm. One-way ANOVA was performed and *p* values were calculated using Tukey’s multiple comparison test; ∗*p <* 0.05, ∗∗*p <* 0.01, ∗∗∗*p <* 0.001.

We next examined the involvement of the Rho GTPase family, a major regulator of actin dynamics,^44^ in ATG9B-mediated actin remodeling. Upon the activation of Rho GTPases, Rho-associated coiled-coil kinase (ROCK) and p21 activation kinase (PAK) become activated to phosphorylate LIM kinase (LIMK), which phosphorylates and inactivates one of the critical regulators of actin depolymerization: actin depolymerization factor/cofilin.^45^^,^^46^ Arp2/3 complex, required to form branched networks of actin filaments^47^ and bacterial internalization,^48^ is also activated downstream of Rho GTPases (Figure 4A). Therefore, we observed the morphology of F-actin in *ATG9B* KD cells treated with a series of inhibitors (Figure 4A). The F-actin aggregation that appeared in *ATG9B* KD cells was abolished by treatment with ROCK inhibitor Y27632, PAK inhibitor G5555, or LIMK inhibitor LIMKi 3, but not by Arp2/3 inhibitor CK666 (Figures 4B and 4C). These results suggested that ATG9B is involved in actin rearrangement through the regulation of the activation of Rock, PAK, or LIMK. To test whether ATG9B interacts with Rho GTPases, we performed a co-immunoprecipitation (co-IP) experiment and found that ATG9B exhibited strong interaction with RhoA and Cdc42, and a slight interaction with Rac1 (Figure 4D). These findings suggested that ATG9B contributes to the activation of these Rho GTPases through interactions. Next, we investigated the activation of these Rho GTPases in *ATG9B* KD cells by pull-down assay with Rhotekin and PAK, which selectively bind to active RhoA and active Rac1 or Cdc42, respectively, using the GTP analog GTPγS as a positive control. If RhoA is activated, its interaction with Rhotekin is stronger, and if Rac1 or Cdc42 is activated, their interactions with PAK are stronger, as with GTPγS.^49^^,^^50^ The activation levels of RhoA, Rac1, and Cdc42 in *ATG9B* KD cells were not predominantly different from those in si-control-treated cells (Figures 4E and S5A–S5C). Next, we examined the phosphorylation of LIMK and cofilin in *ATG9B* KD cells. *p*-LIMK and *p*-cofilin were elevated in *ATG9B* KD cells (Figures 4F, S5D, and S5E). These results suggested that actin depolymerization is suppressed by higher phosphorylation levels of LIMK and cofilin in *ATG9B* KD cells.

### Unc-51-like autophagy-activating kinase 1 is involved in bacterial invasion regulated by ATG9B

**Figure 5:**
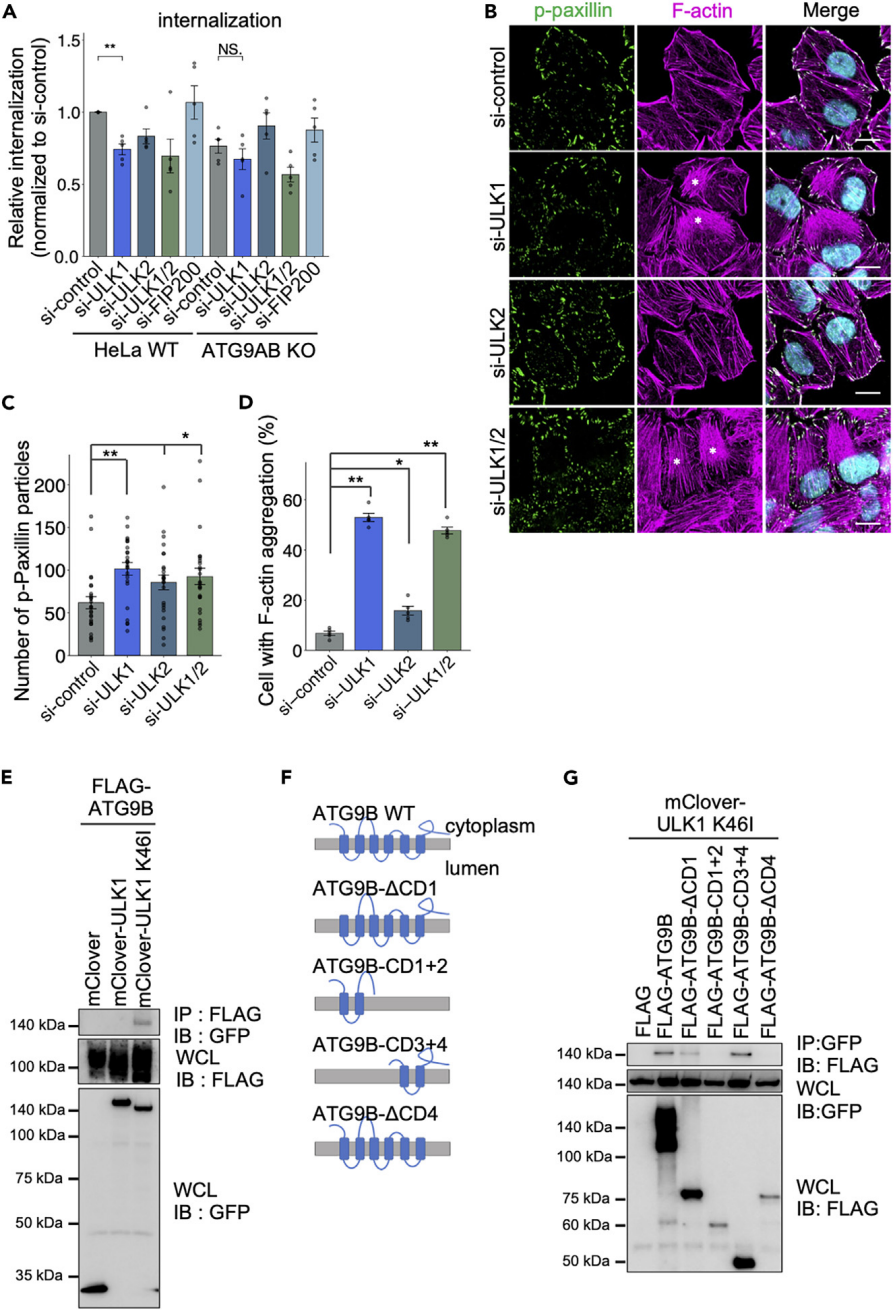
ULK1 is involved in bacterial invasion regulated by ATG9B (A) WT and *ATG9AB* KO HeLa cells were treated with the indicated siRNA and infected with GAS at an MOI of 10. The relative percent internalization was normalized with data from non-targeting control siRNA-treated cells (colony-forming units [CFU] recovered at 2 h post-infection [hpi]/CFUs at 1 hpi). (B–D) Immunostaining of the indicated siRNA-treated, fixed HeLa cells for *p*-Paxillin (green) and F-actin (magenta). Cellular DNA was stained with DAPI (cyan). Asterisks indicate actin aggregation. Representative microscopic images (B) and quantification of the number of *p*-Paxillin particles (C) and percentage of cells with F-actin aggregation.€. (E) Co-IP between FLAG-tagged ATG9B and mClover-tagged ULK1 or ULK1 kinase inactive mutant (K46I) proteins. (F) Schematic representation of the cytoplasmic domain (CD) mutants of ATG9B. (G) Co-IP between the indicated FLAG-tagged ATG9B and mClover-tagged ULK1 KI mutant proteins.Data in c (*n* = 200 cells per condition) and d (*n* = 100 cells per condition) represent the mean ± SEM from five independent experiments. Scale bar, 10 μm. One-way ANOVA was performed and *p* values were calculated using Tukey’s multiple comparison test; ∗*p <* 0.05, ∗∗*p <* 0.01.

The localization of ATG9A is regulated by ULK1 kinase activity.^51^ To determine whether ULK is also involved in ATG9B-regulated bacterial invasion, we performed a gentamicin protection assay using siRNA KD in WT HeLa cells and *ATG9A/B* KO cells. Treatment with *ULK1* siRNA decreased the GAS internalization rate in WT cells but not in *ATG9AB* KO cells (Figures 5A and S6A), suggesting that ULK1 is involved in the ATG9B-mediated internalization of GAS. In contrast, KD of *FIP200*, a component of the ULK1 complex required for autophagy induction, did not change the GAS internalization rates, indicating that the physiological function of ULK1 in bacterial internalization is distinct from that in autophagy.

Next, confocal microscopy showed paxillin and F-actin in cells with *ULK1* and *ULK2* KD. We found that KD of *ULK1* or *ULK2* increased *p*-paxillin particle and aggregation of F-actin, with particularly remarkable effects under *ULK1* KD (Figures 5B–5D). To examine whether ULK1 mediates bacterial invasion through its kinase activity, we complemented the cells with the expression of siRNA-resistant WT ULK1 and a kinase activity-deficient ULK1 mutant (K46I). Ectopic expression of WT ULK1 in *ULK1* KD cells abolished actin aggregation, but that of the K46I mutant did not (Figures S6B and S6C), suggesting that ULK1 is involved in actin rearrangement via its kinase activity.

ATG9A reportedly interacts with ULK1^51^^,^^52^; thus, we performed a co-IP experiment for interaction between ATG9B and ULK1 (Figure 5E). Although ATG9B did not interact with WT UKL1, it did bind to the ULK1 K46I mutant (Figure 5E). ATG9B is phosphorylated by ULK1, but the interaction may be weak or instantaneous. Therefore, the interaction with the ULK1 K46I mutant, rather than with WT ULK1, was confirmed using co-IP. Furthermore, the ectopic expression of ULK1 K461 altered the subcellular localization of ATG9B (Figure S6D). These results suggested that ULK1 interacts with ATG9B to regulate ATG9B localization through phosphorylation, leading us to utilize the ULK1 K46I mutant as a tool to study its interaction with ATG9B.

ATG9B is a multi-transmembrane protein anchored in intracellular vesicles. Therefore, we generated a series of ATG9B cytoplasmic domain (CD)-deficient mutants (Figure 5F) to determine which domains are essential for its interaction with ULK1. The CD4 region of ATG9B was critical for the interaction with ULK1 in co-IP experiments (Figure 5G). To examine whether the CD4 region is required for the ATG9B-mediated regulation of actin remodeling, we expressed siRNA-resistant WT ATG9B and CD4-deficient mutants (ΔCD4) in cells with *ATG9B* KD. Confocal microscopy revealed that the CD4 region was essential for actin rearrangement (Figures S6E and S6F). These results suggested that ULK1 is involved in ATG9B-regulated GAS invasion and actin rearrangement.

## Discussion

Previous reports of ATG proteins have focused on their roles in autophagy induction.

Before this study, little was known about the other physiological functions of ATG proteins, especially during bacterial infection. Here, we screened KOs of ATG genes in HeLa cells for involvement in GAS internalization and identified a role for ATG9B in regulating actin rearrangement through the phosphorylation of LIMK and cofilin.

Initially, *ATG9B* KO cells were used to evaluate the effect of ATG9B on GAS internalization, but *ATG9B* KD cells were found to be more effective for this purpose. It has been reported that, in some cells with the gene KO, genes with similar functions complement the KO gene.^53^^,^^54^ It is possible that one or more genes with functional similarity to *ATG9B* were expressed and served to complement the *ATG9B* KO, thereby mitigating its effects on GAS internalization. Thus, in the evaluation using *ATG9AB* and *ATG9B* KO cells, GAS internalization was reduced by 30%–40%. Alternatively, KD transiently deletes the target gene, allowing for evaluation before putative complementation genes become active, which in this case must have been conducive to bacterial internalization.

Our previous work demonstrated that Beclin1 and UVRAG are involved in bacterial internalization, in which an anti-apoptotic complex of Bcl-xL, Beclin1, and UVRAG, but not PI3P, regulates GAS internalization.^26^^,^^55^ We did not observe F-actin aggregation in *Beclin1* or *UVRAG* KO cells (Figure S7). Therefore, ATG9B may regulate GAS internalization through a different pathway than Beclin1-UVRAG.

Among the core ATG proteins, ATG9 is the only transmembrane protein observed in small vesicles near the Golgi, mitochondria, and the pre-autophagosomal structure (PAS).^56^ ATG9 vesicles are targeted to the PAS, where the initial formation of membranes is thought to be generated using these ATG9 vesicles as a membrane source.^57^ Therefore, we sought to confirm whether ATG9A and ATG9B are critical for autophagy induction during bacterial infection. ATG9B did not affect autophagosome formation during GAS infection in HeLa cells (Figure 2). The low expression of ATG9B in HeLa cells suggested that ATG9A is primarily responsible for the formation of autophagosomes. Indeed, during GAS infection, autophagy receptor proteins and autophagosomes accumulated and acidification within autophagosomes was reduced in *ATG9A* KO cells but not in *ATG9B* KO cells (Figure 2). The involvement of ATG9A in autophagosome maturation has been reported previously.^57^^,^^58^

Recently, ATG9 was shown to be involved in trafficking the integrin β1 subunit to the plasma membrane.^35^^,^^59^ Therefore, the internalization of invasive bacterial pathogens that invade host cells via integrin α5β1, such as GAS and *S. aureus*,^4^^,^^5^ is thought to be regulated by ATG9B. However, the internalization processes of *L. monocytogenes*, which invades host cells using E-cadherin and c-Met as receptors,^2^^,^^3^ and *S. enterica* Typhimurium, which regulates actin rearrangement by injecting virulence factor proteins into host cells,^60^ were also regulated by ATG9B (Figures 3F–3H). Because bacterial internalization is commonly mediated by actin regulation, these results pointed to a role for ATG9B in actin regulation. We found that KD of *ATG9B* resulted in F-actin aggregation, which was shown to be induced by the phosphorylation of LIMK and cofilin. We conducted experiments with two different siRNAs targeting *ATG9B*, and observed different results in the number of *p*-Paxillin particles and actin aggregation (Figures 3B–3D). ATG9B has splicing variants with a shorter N-terminus.^61^ Therefore, the variation in phenotypes with siRNA #2, which targets the N-terminus of ATG9B, might explain the differences. This verification is a subject for future investigation. Cofilin is involved in F-actin depolymerization, but the phosphorylation of LIMK inhibits its depolymerization function. LIMK and cofilin are factors that act downstream of RhoGTPases. However, analysis of the pull-down assay results showed that the activities of RhoA, Rac1, and Cdc42, which regulate LIMK phosphorylation and activation,^45^^,^^46^ were not altered under KD of *ATG9B*. This assay alone may not be suitable for the evaluation of the RhoGTP activation state because GTPγS, which was used as a positive control in the pull-down assay, did not detect as strong an interaction with RhoA as it did with Rac1 and Cdc42 (Figure 4E). For future studies, it will be necessary to explore other evaluation methods. Furthermore, it is possible that other factors are involved in the regulation of LIMK phosphorylation that have not yet been elucidated.

Src- and ULK1-mediated phosphorylation regulate ATG9 trafficking, and the trafficking of integrin β1 by ATG9B was independent of ULK1 kinase.^35^^,^^51^ However, we found that ULK1 is involved in actin aggregation by *ATG9B* KD cells, and we also found that ATG9B and ULK1 interact. This interaction was only observed in the ULK1 kinase activity-dead mutant, suggesting that ATG9B and ULK1 are fleeting and that ATG9B may be phosphorylated during the interaction. Furthermore, *ULK1* KD did not have a definitive effect on bacterial internalization comparable to that of the *ATG9B* KD, although there was a difference in dominance (Figure 5A). Given that ATG9 localization is reportedly regulated by Src in addition to ULK1, it may be necessary to consider the involvement of other factors that regulate ATG9B localization or interaction in bacterial internalization.

ATG9B is essential for embryonic development and exhibits tissue-specific expression, showing an abundance in organs, including the placenta and ovaries, and minimal expression in the testis, lung, liver, muscle, pancreas, and brain.^62^ However, studies have reported that the expression level of ATG9B is involved in tumorigenesis in colon, breast, and liver cancers.^63^^,^^64^^,^^65^ Most of these studies described the loss of function or high expression level of ATG9B in carcinogenesis mechanisms reliant upon autophagic degradation pathways. Here, we have provided the first report of actin aggregation induced by phosphorylated LIMK and cofilin accumulation in *ATG9B* KD cells (Figure 4). These findings suggest that ATG9B may be involved in previously unknown aspects of Rho-GTPase-mediated oncogenesis, but this possibility requires validation in a future study.

In conclusion, we demonstrated that ATG9B regulates the internalization of various bacteria through actin rearrangement. ATG9B regulates the phosphorylation cascade of LIMK/cofilin, which is involved in actin depolymerization, and interacts with the kinase function of ULK1 in its actin rearrangement role.

### Limitations of the study

Our research has elucidated the role of ATG9B in bacterial internalization and demonstrated that ATG9B regulates actin rearrangement through the phosphorylation of LIMK and cofilin. *ATG9B* KO mice are early embryonic lethal. Therefore, for this study, we conducted validation experiments exclusively using *in vitro* models. Nevertheless, the study was mainly performed *in vitro* in HeLa cell lines, although multiple Epithelial cell lines were examined. Future research should include lung organoid models or *in vivo* studies to validate the ATG9B function during bacterial infection. Additionally, the mechanism by which ATG9B controls the phosphorylation level of LIMK remains unclear. Future studies are needed to determine whether ATG9B is involved in the phosphorylation of LIMK through RhoGTPases reported to date, or if ATG9B regulates the phosphorylation of LIMK through a different pathway, which requires further investigation.

## STAR★Methods

### Key resources table

**Table:** 

REAGENT or RESOURCE	SOURCE	IDENTIFIER
**Antibodies**		

Anti-ATG14L	MBL	Cat# M184-3; RRID: AB_10897331
Anti-Beclin1	Cell Signaling Technology	Cat# D40C5;RRID: AB_1903911
Anti-UVRAG	Novus Biologicals	Cat# NBP1-18885; RRID: AB_1625950
Anti-ATG5	Cell Signaling Technology	Cat# 12994; RRID: AB_2630393
Anti-ATG7	Cell Signaling Technology	Cat# 8558; RRID: AB_1083119
Anti-ATG16L1	Cell Signaling Technology	Cat# 8089; RRID: AB_1095032
Anti-LC3B	Abcam	Cat# ab51520; RRID: AB_881429
Anti-NDP52	Cell Signaling Technology	Cat# 60732; RRID: AB_2732810
Anti-OPTN	Abcam	Cat# ab23666; RRID: AB_447598
Anti-p62	Santa Cruz Biotechnology	Cat# sc-28359; RRID: AB_628279
Anti-GAPDH	Santa Cruz Biotechnology	Cat# sc-32233; RRID: AB_627679
Anti-ITGA5	Santa Cruz Biotechnology	Cat# sc-13547; RRID: AB_668053
Anti-ITGB1	Millipore	Cat# MAB1965; RRID: AB_2128061
Anti-Paxillin	Cell Signaling Technology	Cat# 12065; RRID: AB_2797814
Anti-*p*-Paxillin	Novus Biologicals	Cat# NBP2-24459
Anti-FAK	Cell Signaling Technology	Cat# 3285S; RRID: AB_2269034
Anti-*p*-FAK	Cell Signaling Technology	Cat# 8556; RRID: AB_10891442
Anti-β-actin	Cell Signaling Technology	Cat# 4970; RRID: AB_2223172
Anti-cofilin	Santa Cruz Biotechnology	Cat# sc-376476; RRID: AB_1115046
Anti-*p*-cofilin	Santa Cruz Biotechnology	Cat# sc-271923; RRID: AB_10611804
Anti-LIMK	Santa Cruz Biotechnology	Cat# sc-28370; RRID: AB_627885
Anti-*p*-LIMK	Cell Signaling Technology	Cat# 3841; RRID: AB_2136943
Anti-RhoA	Santa Cruz Biotechnology	Cat# sc-418; RRID: AB_628218
Anti-Cdc42	Proteintech	Cat# 10155-1-AP; RRID: AB_2078096
Anti-FLAG	Sigma-Aldrich	Cat# A2220; RRID: 10063035
Anti-GFP	Nacalai Tesque	Cat# 04363-24
Anti-Rabbit IgG, HRP-linked	Jackson ImmunoResearch	Cat# 111-005-003; RRID: AB_2337913
Anti-Mouse IgG, HRP-linked	Jackson ImmunoResearch	Cat# 115-005-003; RRID: AB_2338447
Anti-Streptococcus pyogenes Group A Carbohydrate	Abcam	Cat# ab9191; RRID: AB_307061
Anti-Streptococcus pyogenes Group A Carbohydrate	Abnova	Cat# PAB13831; RRID: AB_10674659
Anti-ITGB1 K20	Santa Cruz Biotechnology	Cat# sc-18887; RRID: AB_627006
Anti-p62	Santa Cruz Biotechnology	Cat# sc-25575; RRID: AB_2302590
Anti-FK2	Nippon Bio-Test Laboratories	Cat# 0918-2; RRID: AB_2893311
Anti-LC3B	Cosmo Bio	Cat# cac-ctb-lc3-2;RRID: AB_10707197
Anti-Rabbit IgG, Alexa Fluor 488 conjugated	Invitrogen	Cat# A11008; RRID: AB_143165
Anti-Rabbit IgG, AlexaFluor Plus 594 conjugated	Invitrogen	Cat# A32754; RRID: AB_2762827
Anti-Mouse IgG, Alexa Fluor 488 conjugated	Invitrogen	Cat# A32723; RRID: AB_2633275
Anti-Mouse IgG, AlexaFluor Plus594 conjugated	Invitrogen	Cat# A32742; RRID: AB_2762825
Anti-Goat IgG, Alexa Fluor 594 conjugated	Molecular Probes	Cat# A-11056; RRID: AB_2534103

**Bacterial and virus strains**		

*Streptococcus pyogenes* strain JRS4 (M6+F1^+^)	Laboratory stock	N/A
*Staphylococcus aureus* strain KUH180129	Hikichi et al.^66^	N/A
*Listeria monocytogenes* strain EGD	Laboratory stock	N/A
*Salmonella enterica* Typhimurium strain LT2	Laboratory stock	N/A
*Escherichia coli* strain DH10B	ThermoFisher Scientific	Cat# EC0113

**Chemicals, peptides, and recombinant proteins**		

Dulbecco’s modified Eagle’s medium (DMEM)	Nacalai Tesque	Cat# 08459-64
Fetal Bovine Serum	SIGMA	Cat# 172012
Gentamicin	Nacalai Tesque	Cat# 16672-04
OPTI MEM	Gibco	Cat# 31985-070
Lipofectamine 3000	Invitrogen	Cat# L3000-015
Puromycin	InvivoGen	Cat# ant-pr-1
DAPI	Nacalai Tesque	Cat# 19178-91
Protease inhibitor cocktail	Nacalai Tesque	Cat# 03969-21
LyosoTracker Red DND-99	Molecular Probes	Cat# L-7528
Alexa Fluor 568-ohalloidin	Invitrogen	Cat# A12380
Y-27632	Nacalai Tesque	Cat# 08945-84
G5555	Cayman	Cat# 21469
CK666	Enzo Life Science	Cat# ALX-270-506-M002
LIMKi3	Tront Research Chemicals	Cat# TRC-L397688

**Critical commercial assays**		

PrimeSTAR mutagenesis basal kit	Takara Bio	Cat# R045A
Rhotekin Rho-binding domain-agarose	Millipore	Cat# 14-383
PAK1 p21-binding domain-agarose	Millipore	Cat# 14-325
PrimeScriptII 1st strand cDNA Synthesis Kit	Takara Bio	Cat# 6210A
SsoAdvanced Universal SYBR Green Supermix	BIO-RAD	Cat# 1725271

**Experimental models: Cell lines**		

HeLa	ATCC	Cat# CCL-2
293T	ATCC	Cat# CRL-3216
A549	ATCC	Cat# CRM-CC1-185
Immortalized Human Bronchial Epithelial Cells (HBEpC)-SV40	abm	Cat# T0486-C

**Oligonucleotides**		

Non-targeting control siRNA #1	Dharmacon	Cat# D-001210-01-01
siATG9A	Ambion	Cat# #1: s35504 and #2: s35505
siATG9B	Ambion	Cat# #1: s50068 and #2: s50069
siULK1	Ambion	Cat# s15964
siULK2	Ambion	Cat# s18705
Primer: qPCR *atg9b* Forward: 5′- CCCCTCATACAAGAAGCTCCC -3′	FASMAC	N/A
Primer: qPCR *atg9b* Reverse: 5′- TGCAGGTTGAGCCTGTGTTG -3′	FASMAC	N/A
Primer: qPCR *gapdh* Forward: 5′- GAGTCAACGGATTTGGTCGT -3′	FASMAC	N/A
Primer: qPCR *gapdh* Reverse: 5′- TTGATTTTGGAGGGATCTCG -3′	FASMAC	N/A

**Recombinant DNA**		

pSpCas9(BB)-2A-Puro (px459) V2.0	Addgene	Cat# #62988
pENTR1A	Invitrogen	Cat# A10462
pcDNA-6.2/N-3xFLAG-DEST	Invitrogen	Cat# 12489027
pcDNA-6.2/N-mClover-DEST	Generated in our laboratory	Nozawa et al.^67^
pShuttle-ATG9B	GeneCopeia	Cat# GC-H9593-CF
pSpCas9(BB)-2A-Puro (PX459) V2.0	Addgene	Cat# #62988

**Software and algorithms**		

Fiji/ImageJ	Schindelin et al.^68^	https://fiji.sc
R Studio	Open source	https://www.rstudio.com/

### Resource availability

#### Lead contact

Further information and requests for resources and reagents should be directed to and will be fulfilled by the lead contact, Dr. Ichiro Nakagawa (nakagawa.ichiro.7w@kyoto-u.ac.jp).

#### Materials availability

This study did not generate new unique reagents.

#### Data and code availability

Data: All data reported in this paper will be shared by the lead contact upon request.
Code: This paper does not report the original code.
Any additional information required to reanalyze the data reported in this paper is available from the lead contact upon request.

### Experimental model and study participant details

#### Cells

HeLa cells, 293-T cells, A549 cells and HBEpC cells were grown in Dulbecco’s modified Eagle’s medium (Nacalai Tesque) supplemented with 10% fetal bovine serum (Gibco) and 50 μg/mL gentamicin (Nacalai Tesque) in a 5% CO_2_ incubator at 37°C.

#### Bacterial strains and infection

GAS strain JRS4 (M6^+^F1^+^) and *S. aureus* strain were grown in Todd-Hewitt broth (BD Diagnostic Systems) supplemented with 0.2% yeast extract. *L. monocytogenes* was grown in brain-heart infusion broth (Sigma-Aldrich). *S. enterica* Typhimurium was grown in Luria-Bertani broth. HeLa cells cultured in media without antibiotics were infected for 1 h at a multiplicity of infection (MOI) of 100 for confocal microscopic analysis and an MOI of 10 for the gentamicin protection assay. Infected cells were washed with phosphate-buffered saline (PBS) and treated with 100 μg mL^˗1^ gentamicin for an appropriate time to kill bacteria that had not been internalized.

### Method details

#### Plasmid construction, gene KD, and cell transfection

Human *ULK1* was amplified by polymerase chain reaction (PCR) from total mRNA derived from HeLa cells and was introduced into pcDNA-6.2/N-3xFLAG-DEST and pcDNA-6.2/N-mClover-DEST using Gateway technology (Invitrogen). *ATG9B* and *ULK1* were mutated by site-directed mutagenesis using a PrimeSTAR mutagenesis basal kit (Takara Bio). Polyethylenimine or Lipofectamine 3000 (Invitrogen) was used to transfect cells with plasmids or siRNAs.

#### Generation of KO cells

HeLa strains with the following gene KOs were previously established in our laboratory: *FIP200*, *ATG13*, and *ATG14L*, *Beclin1*, *UVRAG*, *ATG7*, *ATG5*, *ATG16L1*.^26^^,^^67^ HeLa cell KOs of *ATG9A*, *ATG9B*, and *ATG9AB* were generated here using the CRISPR/Cas9 gene editing system.^69^ Two guide RNAs (gRNAs) for HeLa KO cells were designed and cloned into the pSpCas9(BB)-2A-Puro (px459) V2.0 vector: 5′-CTTCTTCTCTCGAATATCCT-3′ for *ATG9A*, and 5′-CACCCGCGAAGAAAACGAGC-3′ for *ATG9B*. HeLa cells were transfected with the plasmids for 48 h, then cultured in selection medium containing 2 μg/mL puromycin. Single colonies were picked and cultured in 24-well plates; after expanding the cultures, deletion of the target gene was confirmed by immunoblotting and sequencing of the target loci.

#### Fluorescence microscopy

Cells were washed with PBS, fixed for 15 min with 4% paraformaldehyde in PBS, permeabilized with 0.1% Triton X-100 in PBS for 10 min, washed with PBS, and blocked at room temperature for 1 h with 2% bovine serum albumin (BSA) and 0.02% NaN_3_ in PBS. Cells were then probed at 4°C overnight with the primary antibody diluted in blocking solution, washed with PBS, and labeled with the secondary antibody at room temperature for 2–3 h. To visualize bacterial and cellular DNA, cells were stained with 4′,6-diamidino-2-phenylindole (DAPI). Confocal fluorescence micrographs were acquired with an LSM900 laser-scanning microscope with a Plan-Apochromat 63×/1.4 oil DIC objective lens and ZEN software (Carl Zeiss) or an FV1000 laser-scanning microscope with a UPlanSApo 100× oil/1.40 objective lens and Fluoview software (Olympus).

#### Bacterial internalization and viability assays

The MOI conditions for this commonly used assay were the same as those described in previous reports,^22^^,^^23^^,^^24^^,^^25^ with modifications to the timing of antibiotics addition and lysate collection. HeLa cells cultured at a 1 × 10^5^/well density in 24-well plates were infected with bacteria at an MOI of 10 for 1 h at 37°C. Subsequently, cells were washed with PBS and cultured in a medium containing 100 μg/mL gentamicin for 1 h to kill extracellular bacteria before collecting the lysate. At 1, 2 h post-infection, cells were washed with PBS and lysed in sterile distilled water. Serial dilutions of the lysates were plated on tryptic soy broth agar plates. The bacterial internalization rate was calculated as the ratio of intracellular bacteria at 2 h post-infection (hpi) to cell-attached bacteria at 1 hpi. As a control experiment, supernatants of host cells or bacteria cultured in medium containing 100 μg/mL gentamicin for 1 h were also spread on agar plates to confirm that no bacterial colonies would grow (data not shown).

#### Microscopic bacterial internalization assay

HeLa cells were seeded onto coverslips (Matsunami Glass) coated with 0.1% gelatin (BD Diagnostic Systems) in 24-well plates at 2 × 10^4^ cells/well and transfected with 10 pmol siRNA oligonucleotides ATG9A#1, ATG9A#2, ATG9B#1, ATG9B#2, or non-targeting siRNA using Lipofectamine 3000 (Invitrogen) 24 h later. At 48 h post-transfection, cells were infected with GAS at an MOI of 10 for 1 h. Cells were fixed for 15 min with 4% paraformaldehyde in PBS and then washed with PBS. Extracellular bacteria were stained with rabbit anti-GAS (1:500) antibodies at room temperature for 2 h, permeabilized with 0.1% Triton in PBS for 15 min, washed with PBS, and blocked at room temperature for 1 h with 2% BSA and 0.02% NaN_3_ in PBS. Intracellular and extracellular bacteria were stained with goat anti-GAS antibodies (1:500) at 4°C overnight, and anti-rabbit IgG Alexa Fluor 594 (1:500) and anti-goat IgG Alexa Fluor 488 (1:500) antibodies at room temperature for 2–3 h. To visualize bacterial and cellular DNA, samples were stained with DAPI. Confocal fluorescence micrographs were acquired with an FV1000 laser-scanning microscope with a UPlanSApo 100× oil/1.40 objective lens and Fluoview software (Olympus) or an LSM900 laser-scanning microscope with a Plan-Apochromat 63×/1.4 oil DIC objective lens and ZEN software (Carl Zeiss). Red fluorescent images (representing extracellular GAS) were set to pixel intensities of 0–150, followed by the calculation of Pearson’s correlation coefficients as co-localization rates of intracellular + extracellular GAS (Alexa Fluor 488+) and extracellular GAS (Alexa Fluor 594+); higher co-localization rates indicated higher numbers of extracellular GAS. Processing of these images was done using ImageJ/Fiji.

#### Immunoblot analysis

Cell lysate samples were mixed with an equal volume of 2x Laemmli sodium dodecyl sulfate (SDS) sample buffer and boiled for 5 min. Samples were separated by SDS-polyacrylamide gel electrophoresis (PAGE) and then transferred to a polyvinylidene difluoride membrane, which was blocked with 5% skim milk or Blocking One (Nacalai Tesque) and incubated with primary antibody diluted with blocking buffer at 4°C overnight. After washing with PBS containing 0.1% Tween 20 three times, the membrane was incubated with secondary antibody for 2 h at room temperature. After washing three times again, the membrane was reacted with Chemi-Lumi One Super (Nacalai Tesque), and images were obtained using an LAS-4000 mini luminescent image analyzer (Fujifilm). Protein expression was quantified using densitometric analyses (ImageJ).

#### F-actin and G-actin immunoblotting

HeLa cells were seeded in six-well plates at 5.0 × 10^5^ cells/well, then transfection with the indicated siRNA using P3000 Lipofectamine. 48 h later, cells were harvested, washed with PBS, and lysed at room temperature in actin stabilization buffer (50 mM PIPES pH 6.9, 50 mM NaCl, 5 mM MgCl_2_, 5 mM EGTA, 1 mM ATP, 5% glycerol, 0.1% NP-40, 0.1% Triton X-100, 0.1% Tween 20, 0.1% 2-mercaptoethanol) containing proteinase-inhibitor and phosphatase inhibitor cocktails (Nacalai Tesque). Lysates were centrifuged at 100,000 ×g for 60 min at 37°C; the supernatants containing G-actin were recovered, and the pellets containing F-actin were solubilized with actin depolymerization buffer (50 mM PIPES pH 6.9, 50 mM NaCl, 5 mM MgCl_2_, 5 μM cytochalasin D) on ice for 1 h. Aliquots of the supernatant and pellet fractions were separated by SDS-PAGE and then subjected to western blotting with anti-β-actin antibody. Protein expression was quantified using densitometric analyses (ImageJ).

#### Co-IP

HEK293T cells were seeded in six-well plates at 1.0 × 10^6^ cells/well, followed by transfection with the indicated plasmids using polyethylenimine. Two days later, the cells were harvested, washed with PBS, and lysed for 30 min on ice in lysis buffer (10 mM Tris-HCl pH 8.0, 150 mM NaCl, 10 mM MgCl_2_, 1 mM EDTA, 1% Triton X-100) containing a proteinase-inhibitor cocktail (Nacalai Tesque). Lysates were centrifuged at 20,000 ×g for 30 min at 4°C, and the supernatants were incubated with anti-FLAG antibody at 4°C for 2 h with shaking, then with 50% Protein G Sepharose 4B (GE Healthcare Life Sciences) at 4°C for 1 h with shaking. The beads were washed five times with lysis buffer and then analyzed by immunoblotting.

#### Rho GTPase activation assay

HeLa cells were seeded in six-well plates at 5.0 × 10^5^ cells/well, then transfection with the indicated siRNA using P3000 Lipofectamine. Two days later, the cells were harvested, washed with TBS, and lysed using lysis buffer B (50 mM Tris-HCl pH 7.2, 1% Triton X-100, 500 mM NaCl, 10 mM MgCl_2_) containing proteinase-inhibitor and phosphatase inhibitor cocktails (Nacalai Tesque). Lysates were centrifuged at 20,000 ×g for 30 min at 4°C, and the supernatants were incubated with Rhotekin Rho-binding domain-agarose (Millipore) or PAK1 p21-binding domain-agarose (Millipore) at 4°C for 1 h with shaking. The beads were then washed three times with Tris wash buffer (50 mM Tris-HCl pH 7.2, 1% Triton X-100, 150 mM NaCl, 10 mM MgCl_2_) and analyzed by immunoblotting.

#### Reverse transcription quantitative PCR (qPCR)

For gene expression analysis, HeLa cells were transfected with siRNA as described above and the total RNA was isolated using a Quick-RNA Miniprep Kit (Zymo Research). A PrimeScriptII 1st strand cDNA Synthesis Kit (Takara Bio) was used for cDNA synthesis, and specific gene transcripts were quantified using SsoFast EvaGreen Supermix (BIO-RAD). The primer sets used in this study are listed in key resources table. Relative changes in transcription levels upon KD of target genes were calculated using the ΔΔCT method, with values normalized to *GAPDH*.

### Quantification and statistical analysis

Cells containing LC3 or other markers were quantified through direct visualization using a confocal microscope (Olympus or Carl Zeiss). Unless otherwise indicated, 200 GAS-infected cells were examined in each experiment, and at least three independent experiments were performed for each trial. Values, including those plotted, represent the mean ± standard error of the mean (SEM). Pearson’s coefficients were calculated using the JACoP plugin of Fiji/ImageJ software, with manually set thresholds. Western blotting and immunoprecipitation experiments were repeated at least five times, and representative blots are shown. Data were analyzed by one-way ANOVA followed by Dunnett’s test or Tukey’s multiple comparison test.
