# Supplementary material for: ATG9B regulates bacterial internalization via actin rearrangement
Source: iScience. 2024 Apr 10;27(5):109623. doi: 10.1016/j.isci.2024.109623 (PMC11066431; doi:10.1016/j.isci.2024.109623)
Supplement: Document S1. Figures S1–S7 [file mmc1.pdf]

iScience, Volume *27*

Supplemental information

ATG9B regulates bacterial internalization via actin rearrangement

Junpei Iibushi, Takashi Nozawa, Hirotaka Toh, and Ichiro Nakagawa

Figure S1

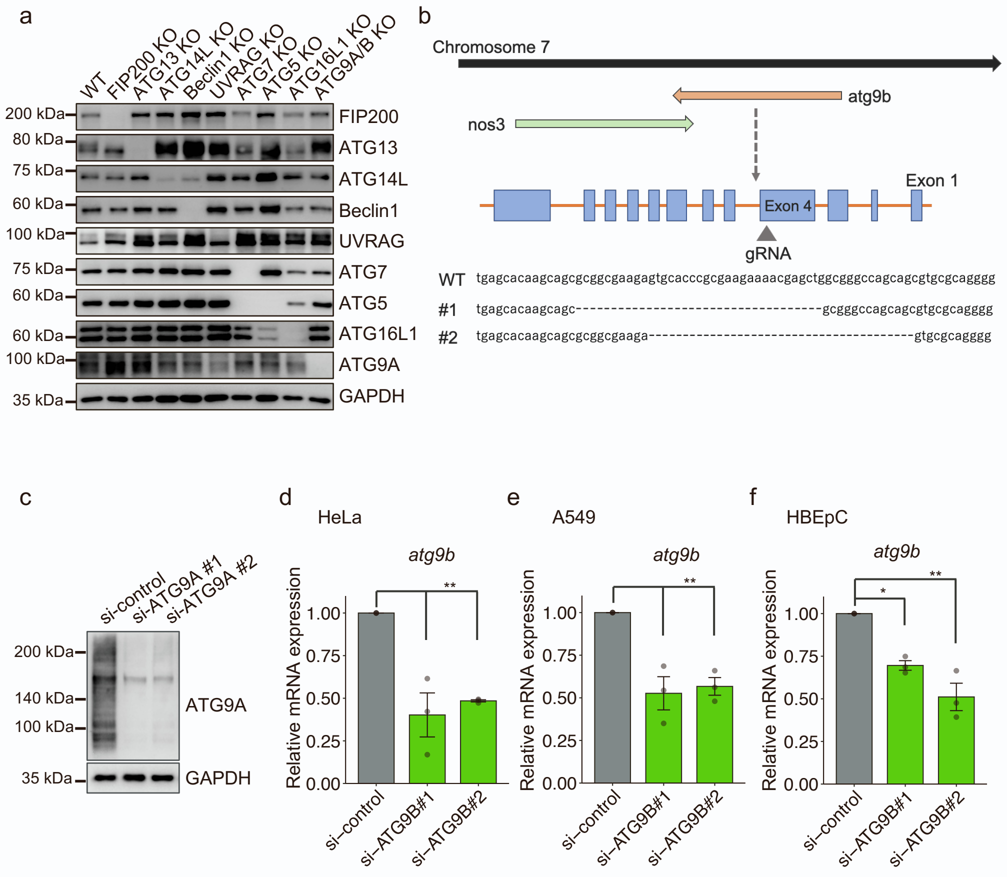

Figure S1. Confirmation of *ATG* knockout and *ATG9A* or *ATG9B* knockdown cells, related to Figure 1.

(a) Immunoblotting analysis of *ATG* KO Hela cell lysates using the indicated antibodies.

(b) Genome sequence of the indicated region in *ATG9B* KO HeLa cells. Genomic DNA purified from *ATG9B* KO HeLa cells, generated by knocking out *ATG9A* using ATG9B #2, was subjected to Sanger sequencing.

(c) Immunoblotting analysis of cell lysates of HeLa cells undergoing a 48-h transfection with the indicated siRNA oligonucleotides (48 h) using an ATG9A-specific antibody.

(d-f) qPCR analysis of *ATG9B* mRNA expression in HeLa cells (d), A549 cells (e), or HBEpCs (f) at 48 h post-transfection with ATG9B siRNA.

Data shown represent individual values and mean ± SEM. *P* values were calculated by one-way ANOVA, followed by Tukey's multiple comparison test; $^{*}P < 0.05$, $^{**}P < 0.01$, $^{***}P < 0.001$.

Figure S2

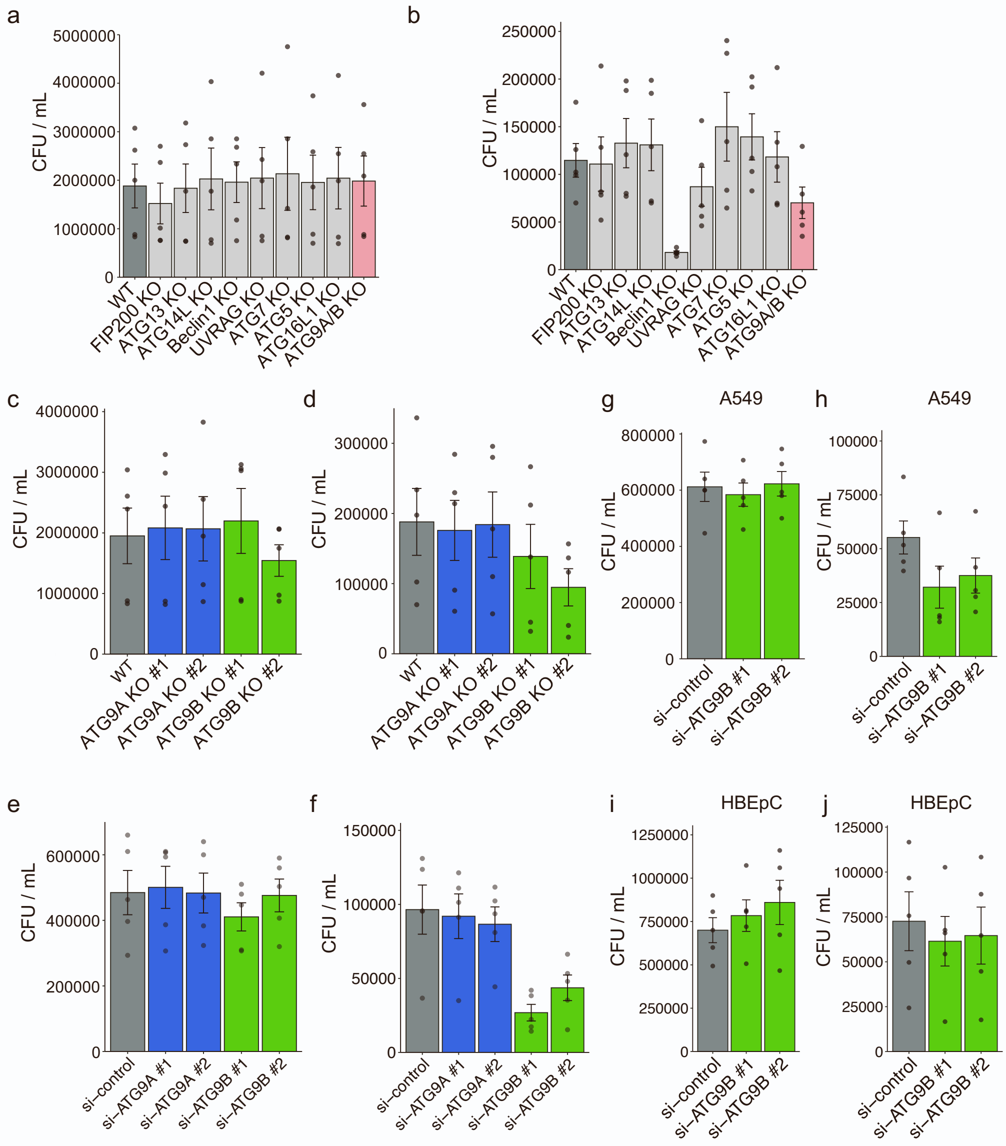

Figure S2. Measurement of CFUs by gentamicin protection assay, related to Figure 1.

(a,b) Hela cells with *ATG* KO were infected with GAS at an MOI of 10 and CFUs were quantified at 1 hpi (a) and 2 hpi (b).

(c–f) *ATG9A*, *ATG9B* KO cells or siRNA treated *ATG9A*, *ATG9B* KD cells were infected with GAS at an MOI of 10, and CFUs were quantified at 1 hpi (c, e) and 2 hpi (d, f).

(g–j) A549 cells (g, h) and HBEpCs (i, j) were treated with ATG9B-targeted siRNA and were infected with GAS at an MOI of 10 and CFUs were quantified at 1 hpi (g, i) and 2 hpi (h, j).

Figure S3

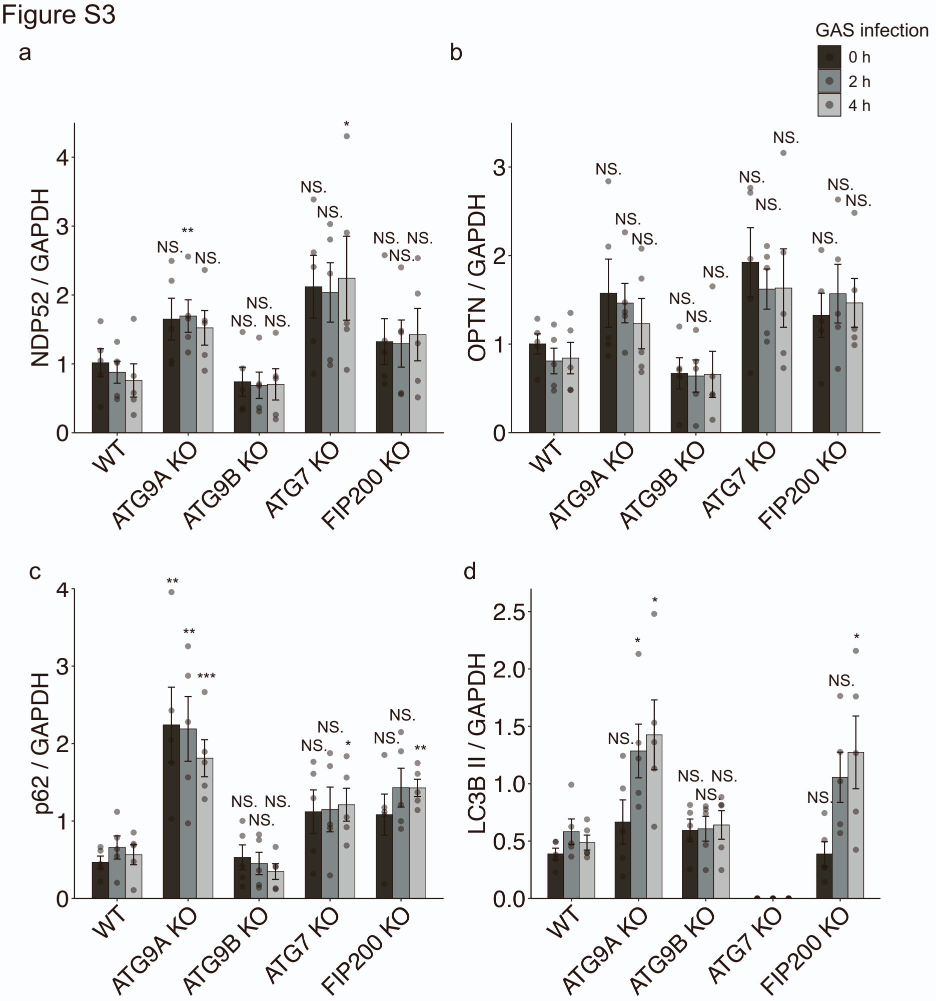

Figure S3. Immunoblot quantification of autophagy receptor and LC3 variation in *ATG9A* and *ATG9B* KO cells during GAS infection, related to Figure 2.

(a–d) HeLa cells with *ATG* KO were infected with GAS for 0 h (non-infection; NI), 2 h, or 4 h. NDP52 (a), OPTN (b), p62 (c), and LC3 (d) protein levels were quantified. GAPDH was used as the loading control. *ATG7* and *FIP200* KO cells were used as a control that cannot be induced into autophagy.

Data shown represent individual values and means ± SEM. *P* values were calculated by one-way ANOVA, followed by Tukey's multiple comparison test; *$P < 0.05$, **$P < 0.01$, ***$P < 0.001$.

Figure S4

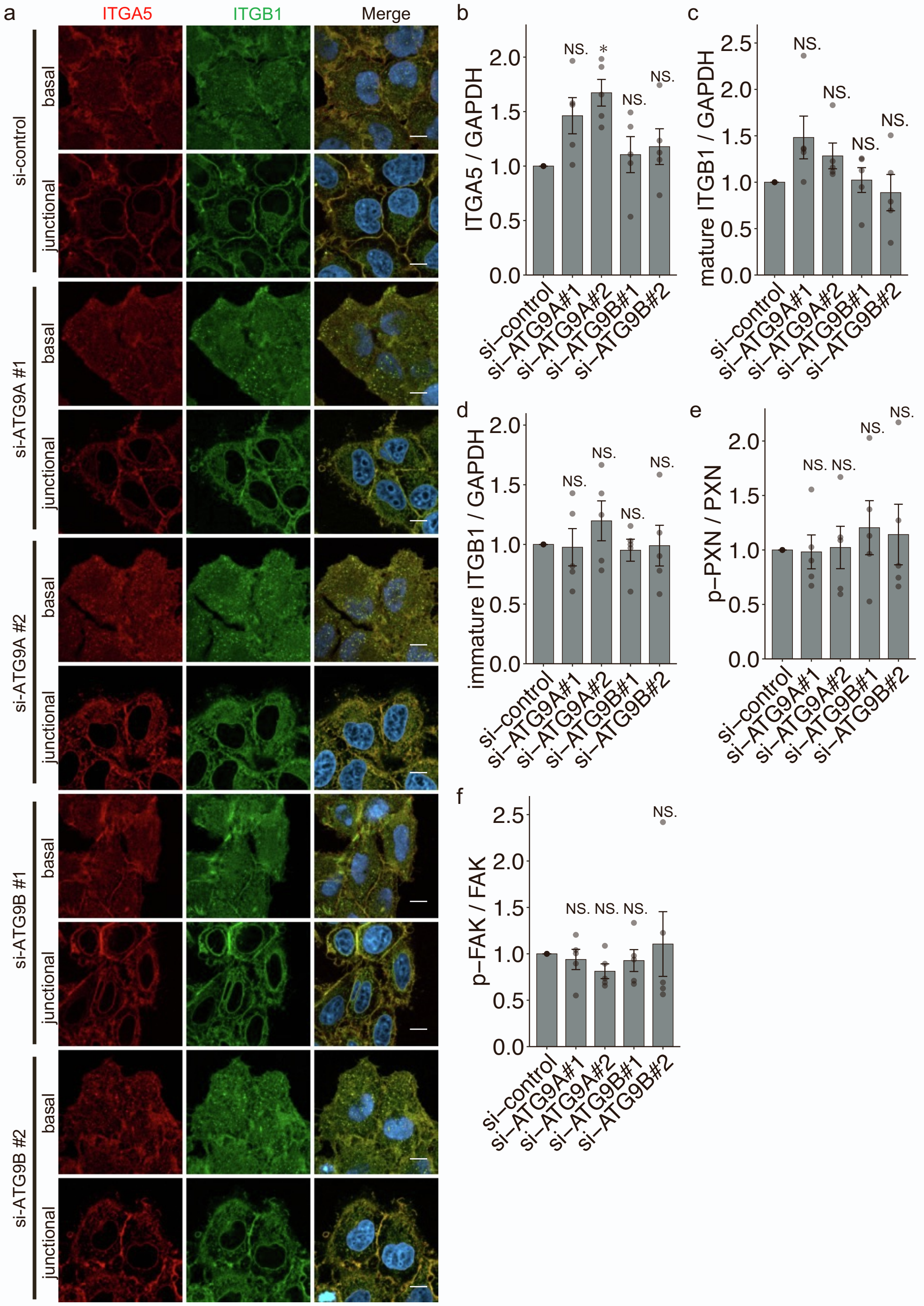

Figure S4. Localization of integrins and immunoblot quantification of focal adhesion factor in *ATG9A* or *ATG9B* KD cells, related to Figure 3.

(a) Immunostaining of fixed, ATG9A, or ATG9B siRNA-treated HeLa cells for integrin α5 (red) and integrin β1 (green). Cellular DNA was stained with DAPI (cyan). Scale bar, 10 μm. (b–e) Quantification of immunoblotting results for focal adhesion factor. ITGA5 (b) and ITGB1 (c) were normalized using GAPDH. p-PXN (d) and p-FAK (e) were quantified using PXN and FAK, respectively.

Data shown represent individual values and means ± SEM. *P* values were calculated by one-way ANOVA, followed by Tukey's multiple comparison test; $*P < 0.05$, $**P < 0.01$, $***P < 0.001$. NS: not significant.

Figure S5

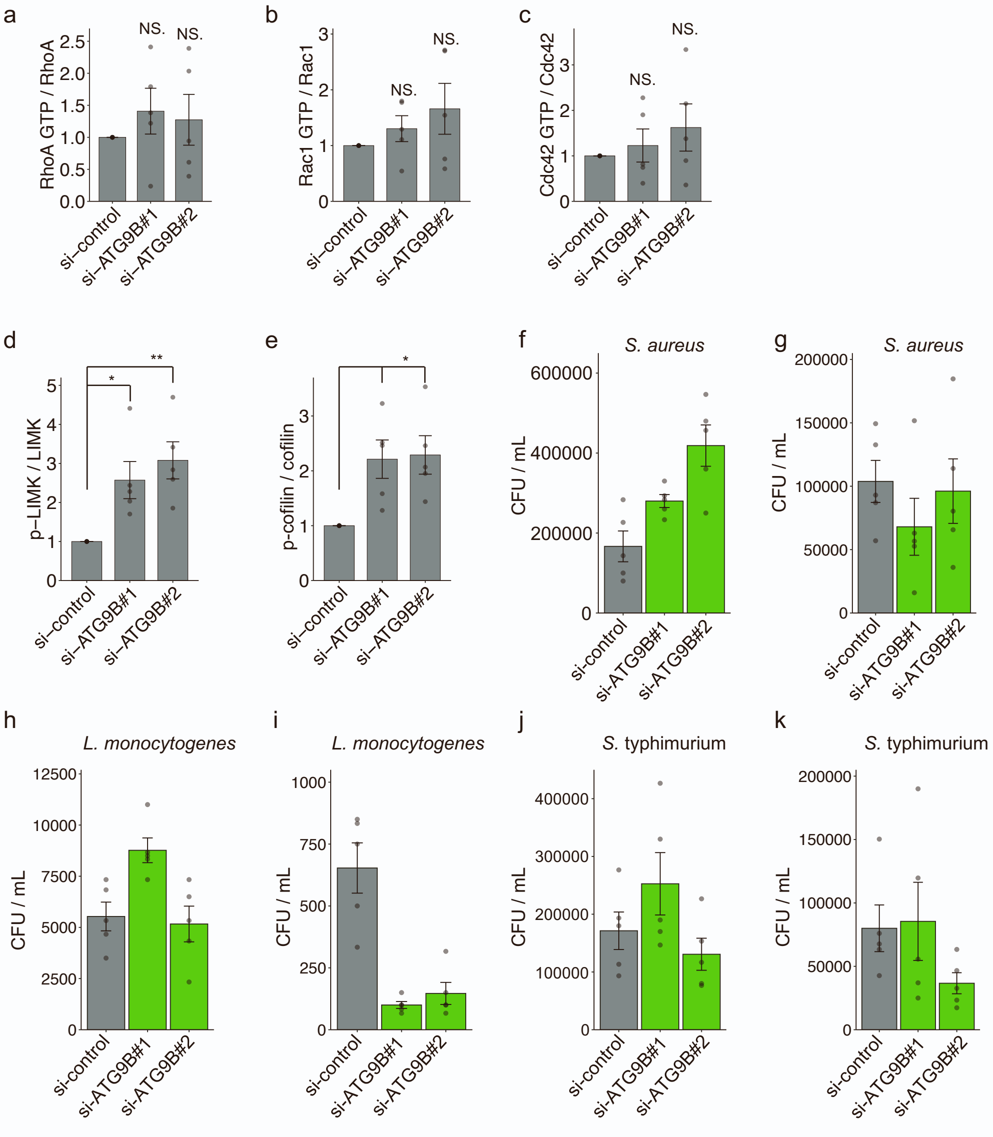

Figure S5. Immunoblot quantification of RhoGTPase activity, p-LIMK and p-cofilin levels, and measurement of bacterial CFUs by gentamicin protection assay, related to Figure 4.

(a–c) Pull-down assay of RhoGTPase activity in *ATG9B* KD cells. Active RhoA (a), Rac1 (b), Cdc42 (c) levels were calculated from the respective RhoGTPase levels in whole cell lysates (WCL).

(d, e) Quantification of protein levels of p-LIMK (d) and p-cofilin (e) using LIMK and cofilin levels, respectively, in *ATG9B* KD cells.

(f–k) HeLa cells treated with ATG9B-targeted siRNA and infected with *S. aureus* (f, g), *L. monocytogenes* (h, i) or *S.* typhimurium (j, k) at an MOI of 10. CFUs were quantified at 1 hpi (f, h, j) and 2 hpi (g, i, k).

Data shown represent individual values and means ± SEM. *P* values were calculated by one-way ANOVA, followed by Tukey's multiple comparison test; *$P < 0.05$, **$P < 0.01$, NS: not significant.

Figure S6

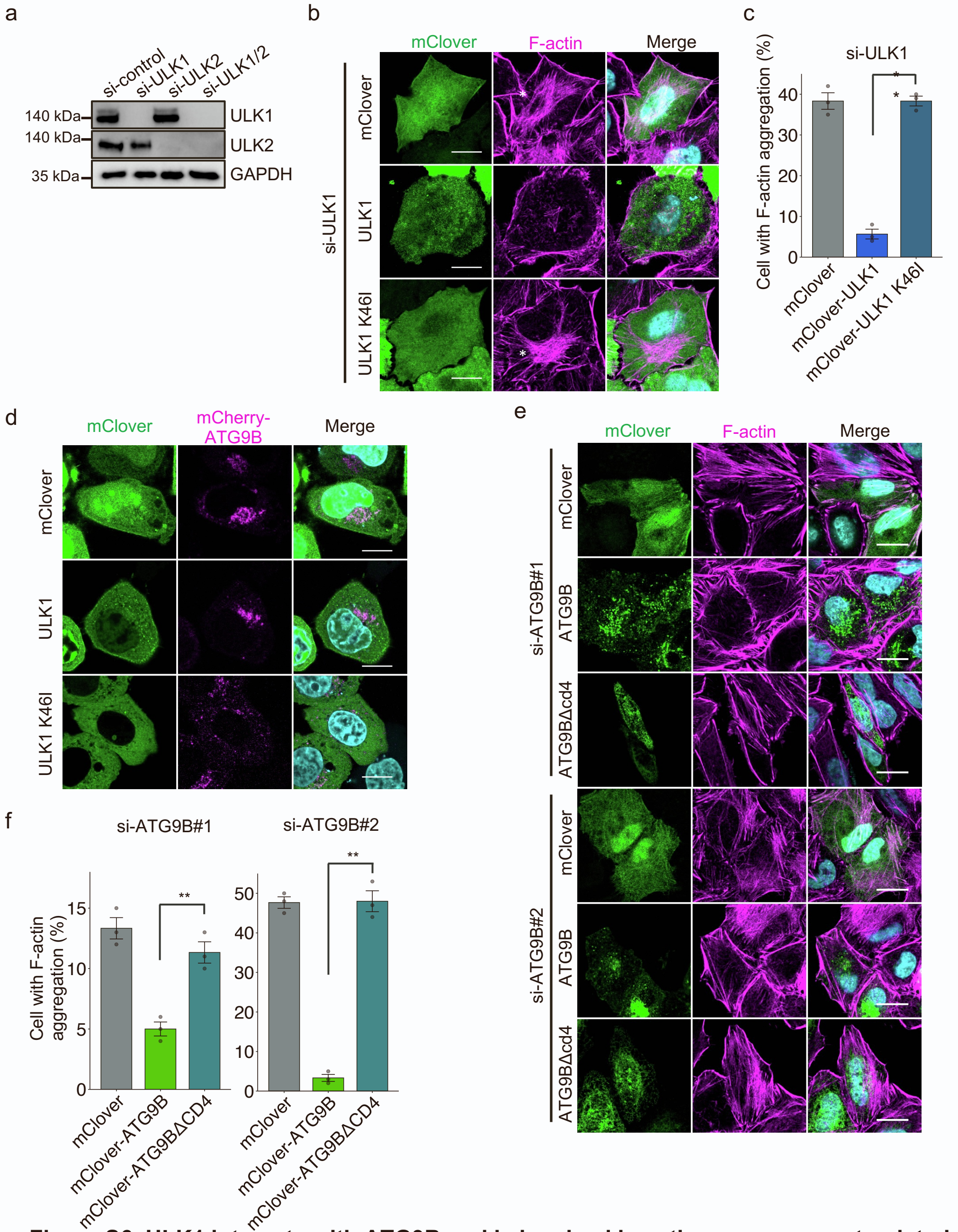

Figure S6. ULK1 interacts with ATG9B and is involved in actin rearrangement, related to Figure 5.

(a) Immunoblotting analysis of lysates of HeLa cells transfected for 48 h with the indicated siRNA oligonucleotides using specific antibodies.

(b, c) ULK1 rescue experiment. HeLa cells transfected with ULK1 siRNA and siRNA-resistant mClover3-tagged ULK1 or ULK1 K46I (green) were fixed and stained for F-actin (magenta). Cellular DNA was stained with DAPI (cyan), and asterisks indicate F-actin aggregation (b). Quantification of the percentage of cells with F-actin aggregation (c).

(d) Cellular localization of ULK1 and ATG9B. HeLa cells were transfected with mClover3-tagged ULK1 or ULK1 K46I (green) and mCherry-tagged ATG9B (magenta) and fixed. Cellular DNA was stained with DAPI (cyan).

(e, f) ATG9B mutant rescue experiment. HeLa cells transfected with ATG9B siRNA and siRNA-resistant mClover3-tagged ATG9B mutants (green) were fixed and stained for F-actin (magenta). Cellular DNA was stained with DAPI (cyan) (e). Quantification of the percentage of cells with F-actin aggregation (f).

Data shown represent individual values and mean ± SEM. Scale bar, 10 µm. *P* values were calculated by one-way ANOVA, followed by Tukey's multiple comparison test; $^{**}P < 0.01$.

Figure S7

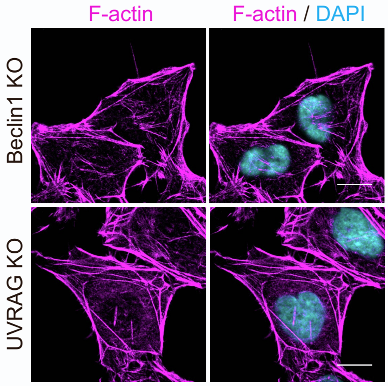

Figure S7. Actin morphology in *Beclin1* and *UVRAG* KO cells, related to discussion section.
Immunostaining of fixed Hela cells with KO of *Beclin1* or *UVRAG* KO cells for F-actin (magenta). Cellular DNA was stained with DAPI (cyan). Scale bar, 10 μm.
